# 6-Shogaol attenuates LPS-induced inflammation in BV2 microglia cells by activating PPAR-γ

**DOI:** 10.18632/oncotarget.16719

**Published:** 2017-03-30

**Authors:** Qinghe Han, Qinghai Yuan, Xiaolin Meng, Junyuan Huo, Yuxin Bao, Guanghong Xie

**Affiliations:** ^1^ The Second Hospital of Jilin University, Changchun 130000, China; ^2^ College of Veterinary Medicine, Jilin University, Changchun 130062, China

**Keywords:** 6-Shogaol, LPS, PPAR-γ, BV2 microglia

## Abstract

6-Shogaol, a pungent agent isolated from *Zingiber officinale Roscoe*, has been known to have anti-tumor and anti-inflammatory effects. However, the anti-inflammatory effects and biological mechanism of 6-Shogaol in LPS-activated BV2 microglia remains largely unknown. In this study, we evaluated the anti-inflammatory effects of 6-Shogaol in LPS-activated BV2 microglia. 6-Shogaol was administrated 1 h before LPS treatment. The production of inflammatory mediators were detected by ELISA. The expression of NF-κB and PPAR-γ were detected by western blot analysis. Our results revealed that 6-Shogaol inhibited LPS-induced TNF-α, IL-1β, IL-6, and PGE2 production in a concentration dependent manner. Furthermore, 6-Shogaol inhibited LPS-induced NF-κB activation by inhibiting phosphorylation and nuclear translocation of NF-κB p65. In addition, 6-Shogaol could increase the expression of PPAR-γ. Moreover, inhibition of PPAR-γ by GW9662 could prevent the inhibition of 6-Shogaol on LPS-induced inflammatory mediator production. In conclusion, 6-Shogaol inhibits LPS-induced inflammation by activating PPAR-γ.

## INTRODUCTION

The incidence of neurodegenerative disease, particularly Parkinson disease (PD) and Alzheimer's disease, increased markedly in the last decades [1, 2]. Microglia, the major immune cells in the brain, plays a key role in host defence response to injury or infectious agents [3]. Microglia is exquisitely sensitive to brain injury and disease [4]. Overactivation of microglia leads to the production of inflammatory mediators which plays a critical role in the development of neuroinflammation [5, 6]. Neuroinflammation has recently been implicated as an important mechanism responsible for the pathological processes of neurodegenerative diseases [7, 8]. Therefore, the identification of agents to inhibit neuroinflammation might be an effective approach for the treatment of neurodegenerative diseases.

6-Shogaol, a pungent agent from *Zingiber officinale Roscoe*, has been reported to have anti-tumor and anti-inflammatory effects. 6-Shogaol has been reported to protect against LPS-induced acute lung injury in mice. Also, 6-Shogaol was found to attenuate neuroinflammation and cognitive deficits in animal models of dementia [9]. Furthermore, 6-Shogaol has been reported to inhibit LPS-induced iNOS and COX-2 expression in macrophages [10]. In addition, studies showed that 6-Shogaol could protect against LPS-induced toxicity in murine astrocytes [11]. However, whether 6-Shogaol could inhibit LPS-induced anti-inflammatory response in activated microglial cells remains unclear. In the present study, we evaluated the anti-inflammatory effects of 6-Shogaol in LPS-stimulated BV2 microglia.

## RESULTS

### Effects of 6-Shogaol on cell viability

**Figure 1:**
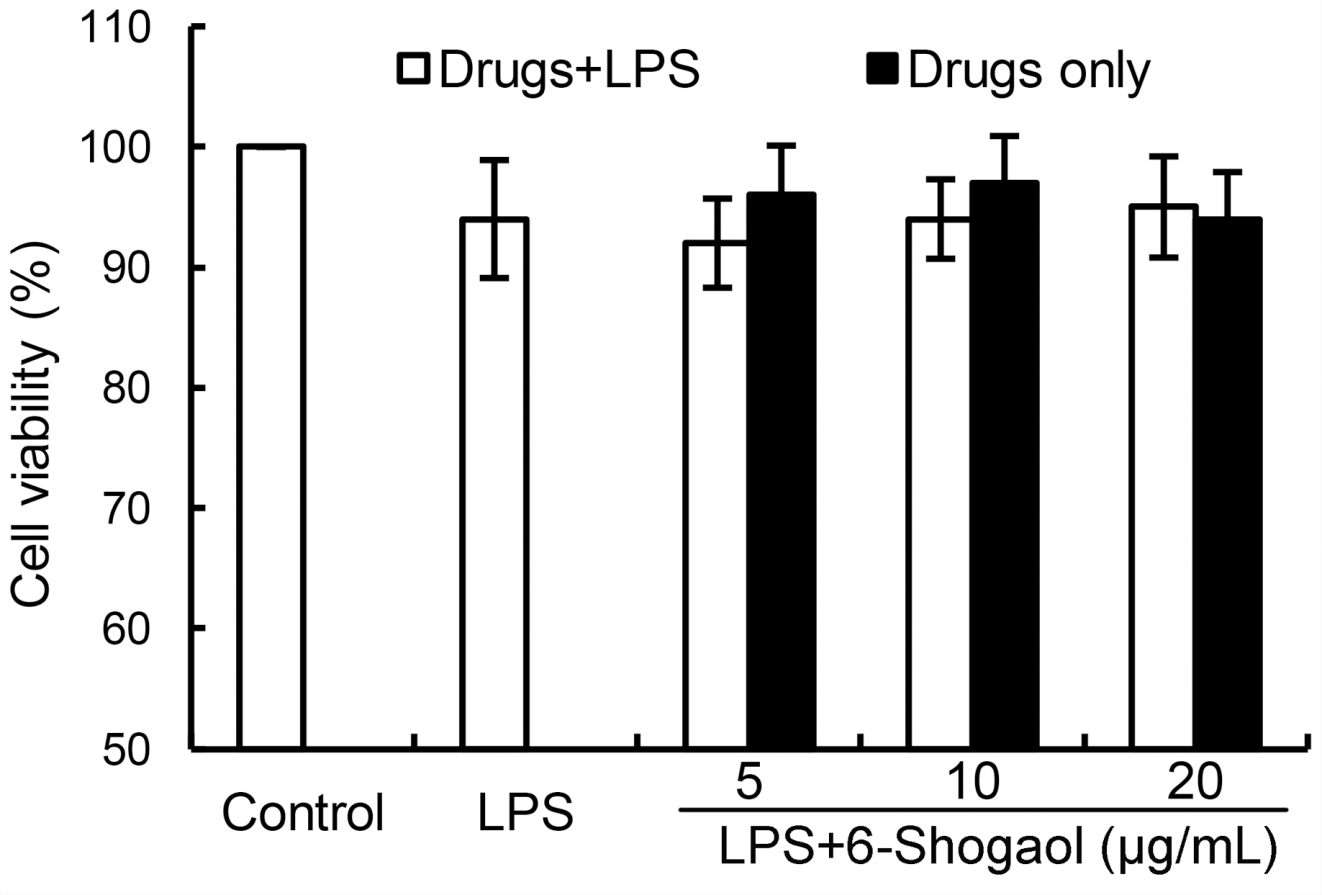
Effects of 6-Shogaol on the cell viability of BV2 microglial cells Cells were cultured with different concentrations of 6-Shogaol (5, 10, 20 μg/ml) in the absence or presence of 0.5 μg/mL LPS for 24 h. The cell viability was determined by MTT assay. The values presented are the means ± SEM of three independent experiments.

To test whether 6-Shogaol has cytotoxicity on BV2 microglia, MTT assay were used in this study. The results showed that 6-Shogaol had no cytotoxicity on BV2 microglia at the concentration of 0 to 20 μg/mL (Figure 1). Therefore, 6-Shogaol (5, 10, 20 μg/mL) were used in the following experiments.

### 6-Shogaol inhibited LPS-induced TNF-α, IL-1ß, IL-6, and PGE_2_ production

**Figure 2:**
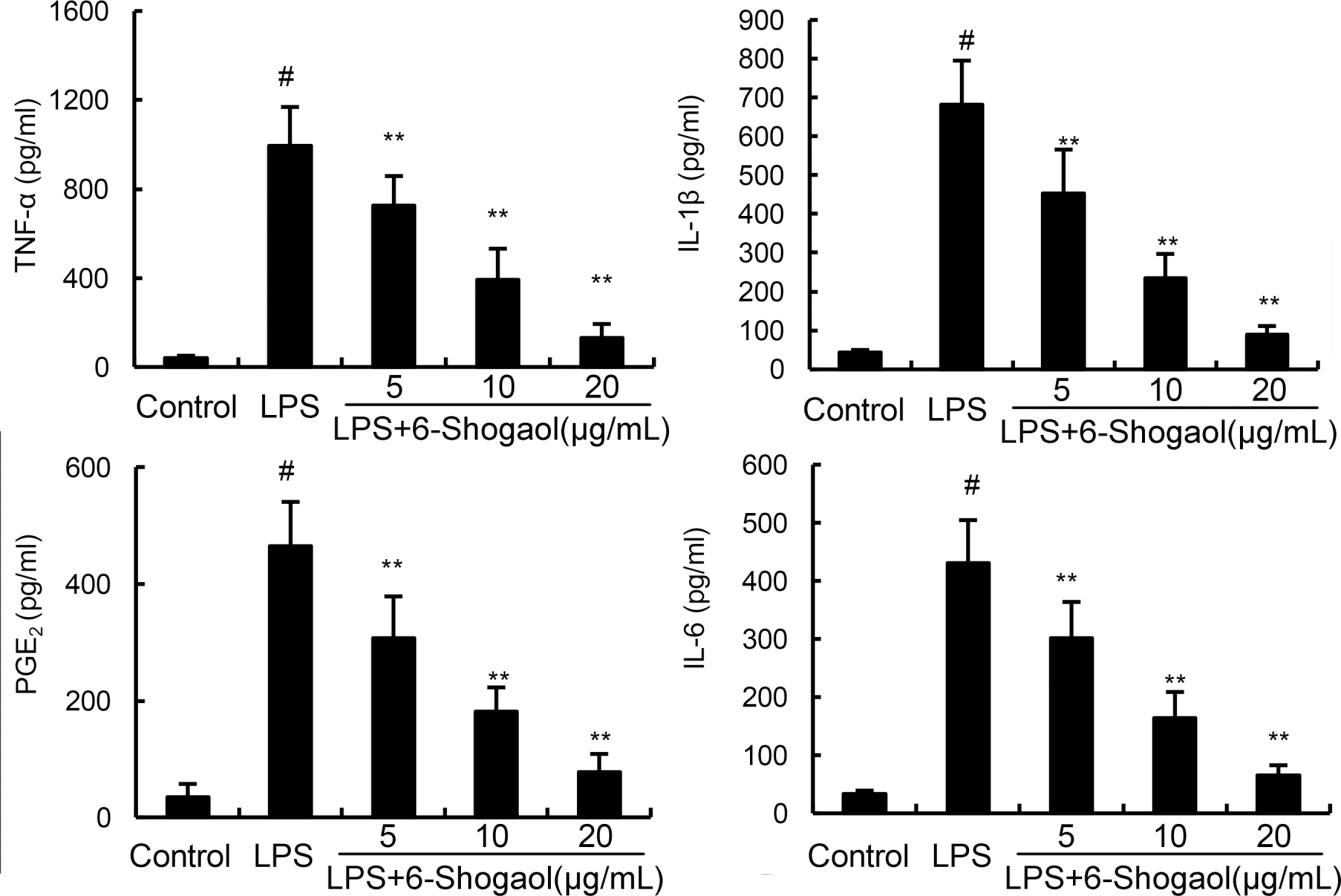
Effects of 6-Shogaol on LPS-induced TNF-α, IL-1ß, IL-6 and PGE_2_ production The production of TNF-α, IL-1ß, IL-6 and PGE_2_ were measured by ELISA. The data presented are the means ± SEM of three independent experiments. ^#^*p* < 0.05 *vs*. control group; *p < 0.05, **p < 0.01 *vs*. LPS group.

To investigate the anti-inflammatory effects of 6-Shogaol, the expression of inflammatory mediators were detected in this study by ELISA. As shown in Figure 2, LPS dramatically increased the production of TNF-α, IL-1ß, IL-6, and PGE_2_. However, 6-Shogaol concentration dependently down-regulated the production of TNF-α, IL-1ß, IL-6, and PGE_2_ induced by LPS.

### 6-Shogaol inhibited LPS-induced NF-κB activation

**Figure 3:**
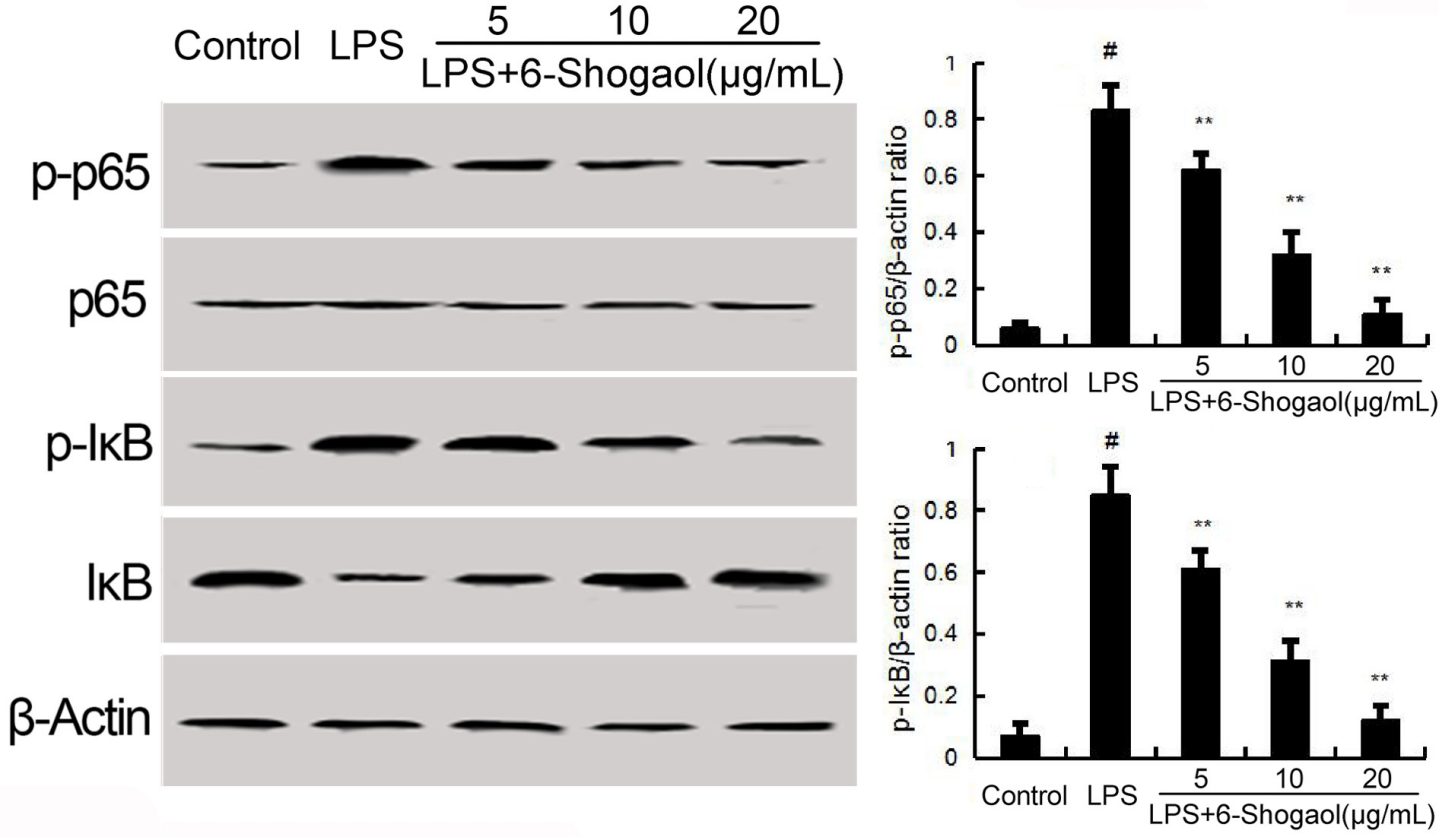
Effects of 6-Shogaol on NF-κB expression The values presented are the means ± SEM of three independent experiments. ^#^*p* < 0.05 *vs*. control group; *p < 0.05, **p < 0.01 *vs*. LPS group.

NF-κB has been known to be involved in the regulation of inflammatory mediators. To investigate the anti-inflammatory mechanism of 6-Shogaol, LPS-induced NF-κB activation were detected in the present study. The results showed that LPS significantly up-regulated the phosphorylation levels of NF-κB p65 and IκBα. Pretreatment of 6-Shogaol concentration dependently inhibited LPS-induced NF-κB p65 phosphorylation and IκBα phosphorylation and degradation (Figure 3).

### Effects of 6-Shogaol on PPAR-γ expression

**Figure 4:**
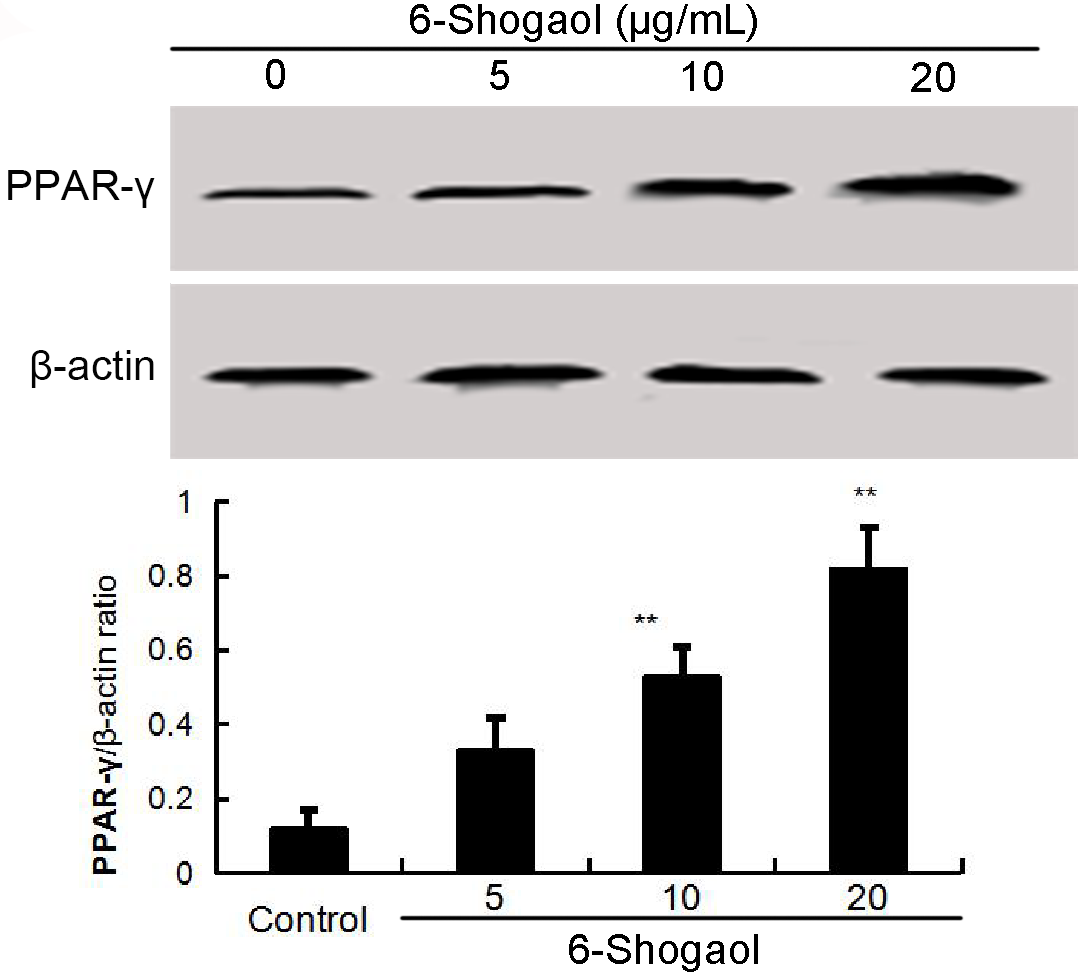
Effects of 6-Shogaol on PPAR-γ expression The values presented are the means ± SEM of three independent experiments. ^#^*p* < 0.05 *vs*. control group; *p < 0.05, **p < 0.01 *vs*. LPS group.

Previous studies showed that activation PPAR-γ could inhibit LPS-induced NF-κB activation. Thus, we detected whether 6-Shogaol could up-regulated the expression of PPAR-γ. As shown in Figure 4, 6-Shogaol increased the expression of PPAR-γ in a concentration dependent manner (Figure 4).

### GW9662 prevented the anti-inflammatory effects of 6-Shogaol

**Figure 5:**
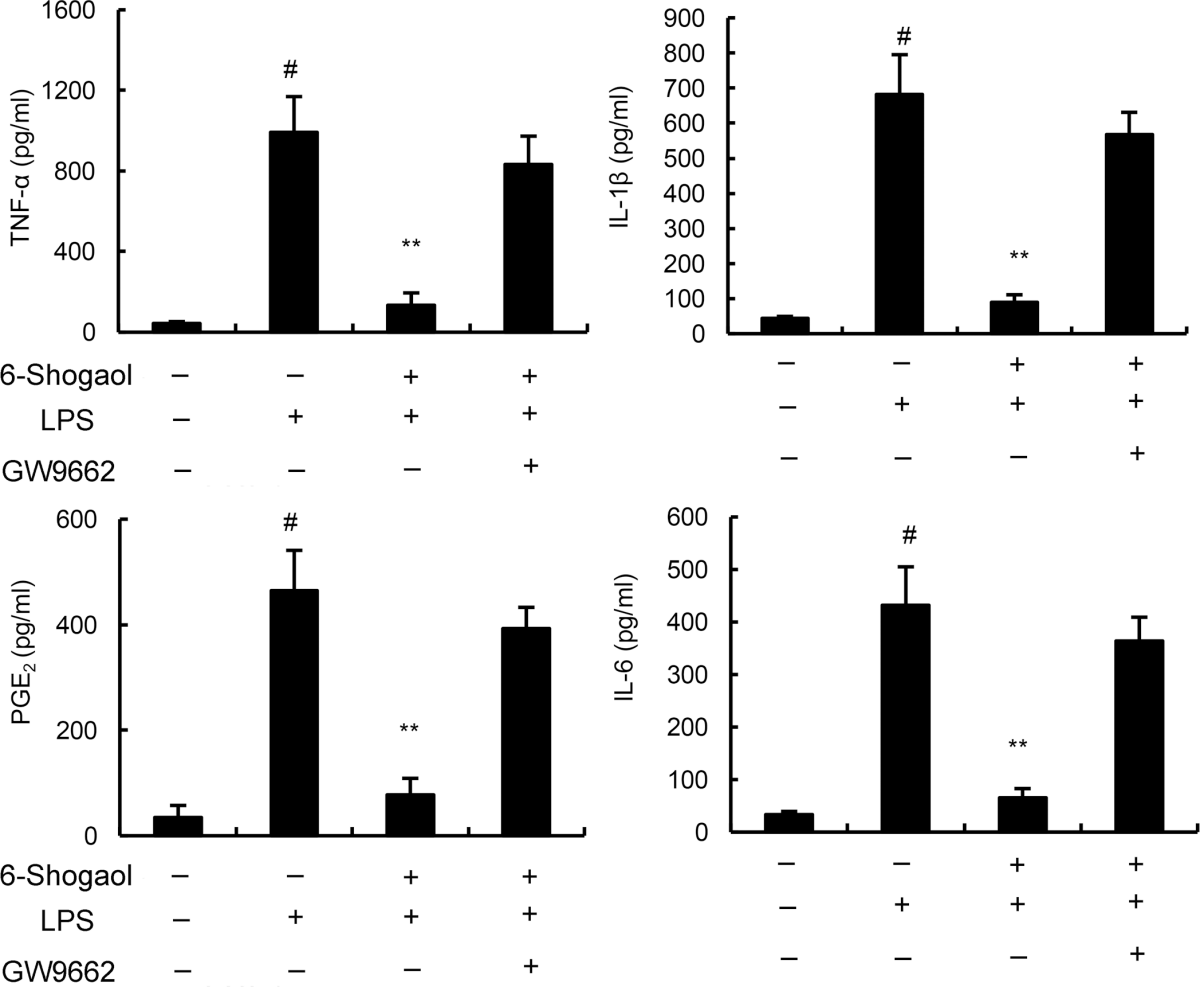
Effects of PPAR-γ inhibitor GW9662 on the anti-inflammatory effects of 6-Shogaol Cells were treated with GW9662 for 12 h. Then, the cells were treated with 6-Shogaol and stimulated by LPS. The productions of inflammatory mediator were detected 24 h after LPS treatment. The values presented are the means ± SEM of three independent experiments. ^#^*p* < 0.05 *vs*. control group; *p < 0.05, **p < 0.01 *vs*. LPS group.

To further evaluate the anti-inflammatory mechanism of 6-Shogaol, PPAR-γ was blocked by its inhibitor GW9662. As shown in Figure 5, our results indicated that the inhibition of 6-Shogaol on TNF-α, IL-1ß, IL-6 and PGE_2_ production were prevented by GW9662. These results suggested that 6-Shogaol exhibited anti-inflammatory effects in BV2 microglia by activating PPAR-γ.

## DISCUSSION

Microglia has been known to play an important role in neurodegenerative diseases [12]. Increasing evidences suggested that controlling the activation of microglia may have protective effects against neurodegenerative diseases [13]. In this study, the results showed that 6-Shogaol inhibited LPS-induced microglia activation by activating PPAR-γ.

Microglia, the prime effector cells in the brain, plays a critical role in immune defense and inflammatory responses [14]. However, overactivation of microglia could lead to the pathological process of neurodegenerative diseases [15]. LPS has the ability to induce microglia activation, which lead to the release of inflammatory mediators [12, 16]. These inflammatory mediators, such as TNF-α, IL-1ß, IL-6 and PGE_2_, play an important role in the pathological process of neurodegenerative diseases [17]. In the present study, our results showed that 6-Shogaol significantly inhibited LPS-induced inflammatory mediators production in BV2 microglia. The results indicated that 6-Shogaol exhibited anti-inflammatory effects in BV2 microglia.

It has been reported that NF-κB played a critical role in neuroinflammation [18]. LPS could induce NF-κB activation and inflammatory cytokines release [19]. Inhibition of LPS-induced NF-κB activation could attenuate neuroinflammation [20]. To clarify the anti-inflammatory mechanism of 6-Shogaol, NF-κB activation were measured in this study. We demonstrated that 6-Shogaol significantly inhibited LPS-induced NF-κB activation. PPAR-γ, belongs to a nuclear receptor superfamily, is a ligand-activated transcription factor [21]. Activation of PPAR-γ could regulate metabolism and inflammation [22]. Previous studies PPAR-γ agonists inhibited LPS-induced airway inflammation [23]. Also, PPAR-γ agonists could suppress LPS-induced inflammatory response in RAW264.7 cells [24]. Furthermore, PPAR-γ agonists have been reported to have therapeutic role in diabetes, inflammation, and cancer [25]. In this study, our results showed that 6-Shogaol increased the expression of PPAR-γ. And GW9662, a PPAR-γ inhibitor, could prevent the inhibition of 6-Shogaol on TNF-α, IL-1ß, IL-6 and PGE_2_ production. These results indicated that 6-Shogaol exhibited anti-inflammatory effects in BV2 microglia by activating PPAR-γ.

In conclusion, our results demonstrated that 6-Shogaol suppressed LPS-induced inflammatory mediators production by activating PPAR-γ, which subsequently inhibited LPS-induced NF-κB activation. 6-Shogaol might be an effective agent for the treatment of neurodegenerative diseases.

## MATERIALS AND METHODS

### Materials

6-Shogaol (purity > 98%) was obtained from the National Institute for the Control of Pharmaceutical and Biological Products (Beijing, China). LPS (Escherichia coli O55:B5) and MTT were purchased from Sigma (St. Louis, MO, USA). ELISA kits of PGE_2_, TNF-α, IL-6 and IL-1β were purchased from BioLegend (San Diego, CA). PPAR-γ monoclonal antibody was obtained from Santa Cruz Biotechnology (Heidelberg, Germany). NF-κB p65, IκBα, and β-actin monoclonal antibodies were obtained from Cell Signaling Technology Inc (Boston, MA, USA).

### Cell culture

Murine BV2 microglia cells were purchased from China Center for Type Culture Collection (CCTCC, Wuhan, China). The cells were cultured in DMEM with 5% fetal bovine serum, 100 U/ml penicillin, and 100 mg/ml streptomycin. The cells were treated with 6-Shogaol 1 h before LPS treatment.

### Cell viability

For determination of cell viability, MTT assay was applied in this study. BV2 microglia was incubated with 6-Shogaol alone and with LPS for 18 h. Then, the cells were treated with MTT for 4 h and the formazan formed was dissolved with DMSO (150 μl/well). The optical density was determined at 570 nm using a Bio-Rad spectrophotometer.

### ELISA assay

24 h after LPS treatment, the levels of TNF-α, IL-1β, IL-6, and PGE2 in culture media were tested using commercially available ELISA kits (BioLegend, San Diego, CA). The assay was performed following the instructions provided by the manufacturers.

### Western blot analysis

The cells were lysed using RIAP lysis buffer and the concentration was measured by BCA method. Equal amount of protein was resolved using 12% SDS-polyacrylamide gel. The proteins were transferred onto PVDF membranes. The membranes were blocked with 5% skimmed milk and incubated with primary antibodies and HRP-conjugated goat anti-rabbit IgG. The proteins were tested using the chemiluminescence detection system (Amersham, Berkshire, UK). Finally, the bands were analyzed using ImageJ software.

### Statistical analysis

Data were presented as means ± SEM. Statistical comparison of the data were analyzed by one-way ANOVA with post-test Neuman-Keuls. A *p value* < 0.05 was considered as significant.

## References

[1] Tanzi RE, Bertram L (2005). Twenty years of the Alzheimer's disease amyloid hypothesis: a genetic perspective. Cell.

[2] Driver JA, Logroscino G, Gaziano JM, Kurth T (2009). Incidence and remaining lifetime risk of Parkinson disease in advanced age. Neurology.

[3] Liu B, Hong JS (2003). Role of microglia in inflammation-mediated neurodegenerative diseases: mechanisms and strategies for therapeutic intervention. J Pharmacol Exp Ther.

[4] Rock RB, Gekker G, Hu S, Sheng WS, Cheeran M, Lokensgard JR, Peterson PK (2004). Role of microglia in central nervous system infections. Clinical microbiology reviews.

[5] Hanisch UK (2002). Microglia as a source and target of cytokines. Glia.

[6] Block ML, Hong JS (2005). Microglia and inflammation-mediated neurodegeneration: Multiple triggers with a common mechanism. Prog Neurobiol.

[7] Glass CK, Saijo K, Winner B, Marchetto MC, Gage FH (2010). Mechanisms Underlying Inflammation in Neurodegeneration. Cell.

[8] Zipp F, Aktas O (2006). The brain as a target of inflammation: common pathways link inflammatory and neurodegenerative diseases. Trends Neurosci.

[9] Moon M, Kim HG, Choi JG, Oh H, Lee PK, Ha SK, Kim SY, Park Y, Huh Y, Oh MS (2014). 6-Shogaol, an active constituent of ginger, attenuates neuroinflammation and cognitive deficits in animal models of dementia. Biochem Biophys Res Commun.

[10] Pan MH, Hsieh MC, Hsu PC, Ho SY, Lai CS, Wu H, Sang SM, Ho CT (2008). 6-Shogaol suppressed lipopolysaccharide-induced up-expression of iNOS and COX-2 in murine macrophages. Mol Nutr Food Res.

[11] Shim S, Kim S, Kwon YB, Kwon J (2012). Protection by [6]-shogaol against lipopolysaccharide-induced toxicity in murine astrocytes is related to production of brain-derived neurotrophic factor. Food Chem Toxicol.

[12] Dheen ST, Kaur C, Ling EA (2007). Microglial activation and its implications in the brain diseases. Curr Med Chem.

[13] Liu B, Hong JS (2003). Role of microglia in inflammation-mediated neurodegenerative diseases: Mechanisms and strategies for therapeutic intervention. J Pharmacol Exp Ther.

[14] Glezer I, Simard AR, Rivest S (2007). Neuroprotective role of the innate immune system by microglia. Neuroscience.

[15] Block ML, Zecca L, Hong JS (2007). Microglia-mediated neurotoxicity: uncovering the molecular mechanisms. Nat Rev Neurosci.

[16] Wang MJ, Lin WW, Chen HL, Chang YH, Ou HC, Kuo JS, Hong JS, Jeng KC (2002). Silymarin protects dopaminergic neurons against lipopolysaccharide-induced neurotoxicity by inhibiting microglia activation. Eur J Neurosci.

[17] Smith JA, Das A, Ray SK, Banik NL (2012). Role of pro-inflammatory cytokines released from microglia in neurodegenerative diseases. Brain Res Bull.

[18] Dilshara MG, Jayasooriya RG, Lee S, Choi YH, Kim GY (2016). Morin downregulates nitric oxide and prostaglandin E2 production in LPS-stimulated BV2 microglial cells by suppressing NF-kappaB activity and activating HO-1 induction. Environmental toxicology and pharmacology.

[19] Covert MW, Leung TH, Gaston JE, Baltimore D (2005). Achieving stability of lipopolysaccharide-induced NF-kappaB activation. Science.

[20] Wang YP, Wu Y, Li LY, Zheng J, Liu RG, Zhou JP, Yuan SY, Shang Y, Yao SL (2011). Aspirin-triggered lipoxin A4 attenuates LPS-induced pro-inflammatory responses by inhibiting activation of NF-kappaB and MAPKs in BV-2 microglial cells. Journal of neuroinflammation.

[21] Yessoufou A, Wahli W (2010). Multifaceted roles of peroxisome proliferator-activated receptors (PPARs) at the cellular and whole organism levels. Swiss Med Wkly.

[22] Chawla A, Barak Y, Nagy L, Liao D, Tontonoz P, Evans RM (2001). PPAR-gamma dependent and independent effects on macrophage-gene expression in lipid metabolism and inflammation. Nat Med.

[23] Birrell MA, Patel HJ, McCluskie K, Wong S, Leonard T, Yacoub MH, Belvisi MG (2004). PPAR-gamma agonists as therapy for diseases involving airway neutrophilia. Eur Respir J.

[24] Huang C, Yang Y, Li WX, Wu XQ, Li XF, Ma TT, Zhang L, Meng XM, Li J (2015). Hyperin attenuates inflammation by activating PPAR-gamma in mice with acute liver injury (ALI) and LPS-induced RAW264.7 cells. Int Immunopharmacol.

[25] Murphy GJ, Holder JC (2000). PPAR-gamma agonists: therapeutic role in diabetes, inflammation and cancer. Trends Pharmacol Sci.

