# Correction: 6-Shogaol attenuates LPS-induced inflammation in BV2 microglia cells by activating PPAR-γ

**DOI:** 10.18632/oncotarget.25638

**Published:** 2018-06-08

**Authors:** Qinghe Han, Qinghai Yuan, Guanghong Xie

**Affiliations:** ^1^ College of Veterinary Medicine, Jilin University, Changchun 130062, China; ^2^ The Second Hospital of Jilin University, Changchun 130000, China

**This article has been corrected:** The correct author infonnation and affiliations are given below:

**Qinghe Han^2^, Qinghai Yuan^2^ and Guanghong Xie^1^**

^1^ College of Veterinary Medicine, Jilin University, Changchun 130062, China

^2^ The Second Hospital of Jilin University, Changchun 130000, China

Original article: Oncotarget. 2017; 8:42001-42006. https://doi.org/10.18632/oncotarget.16719

